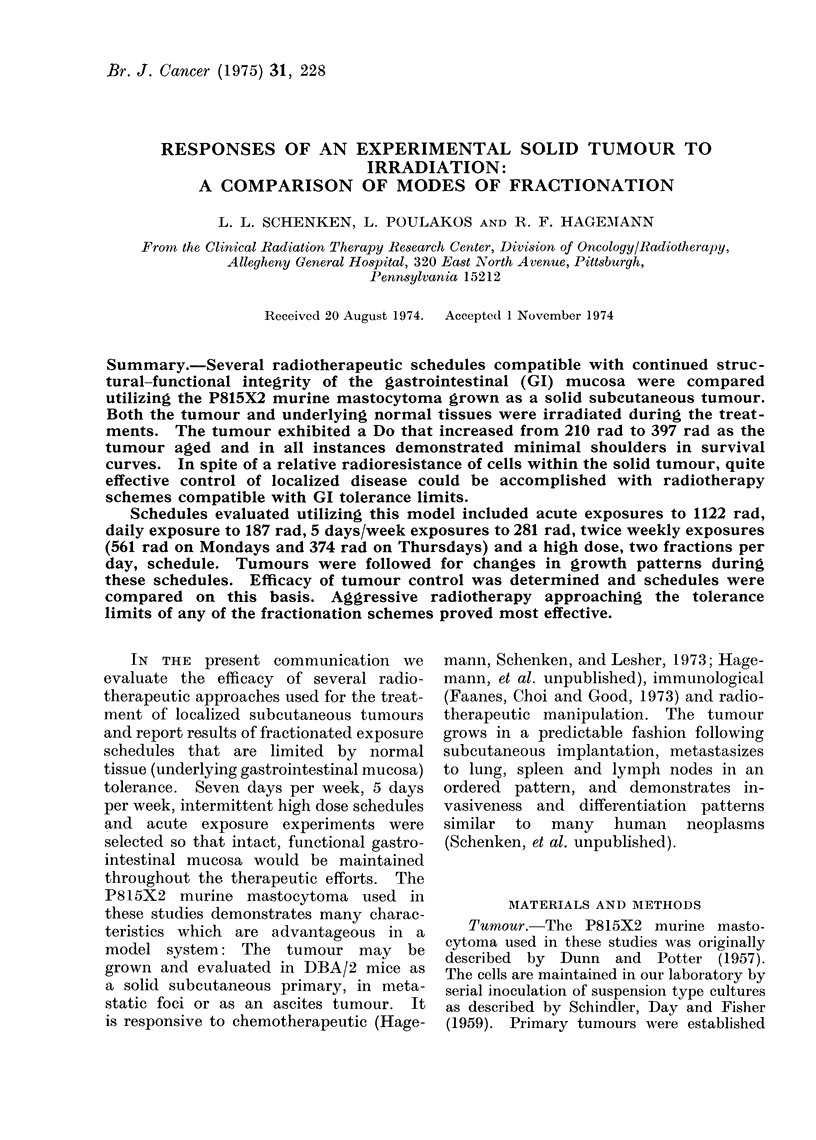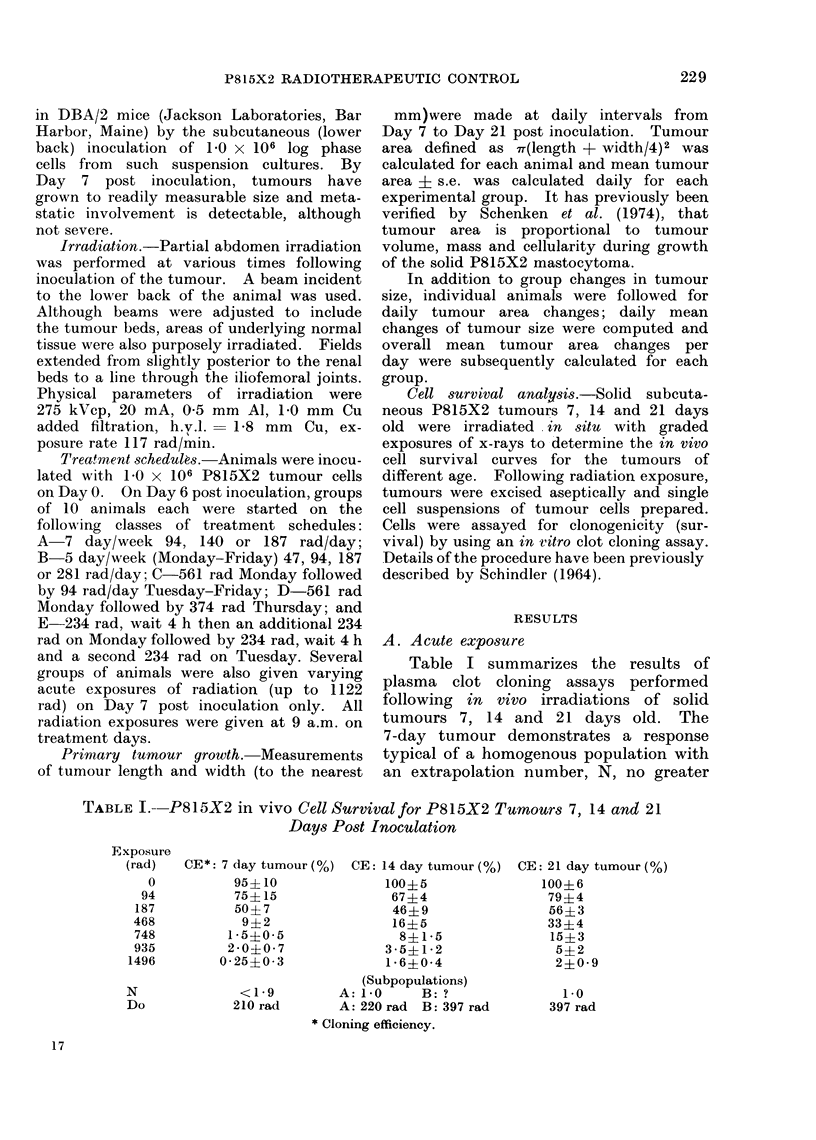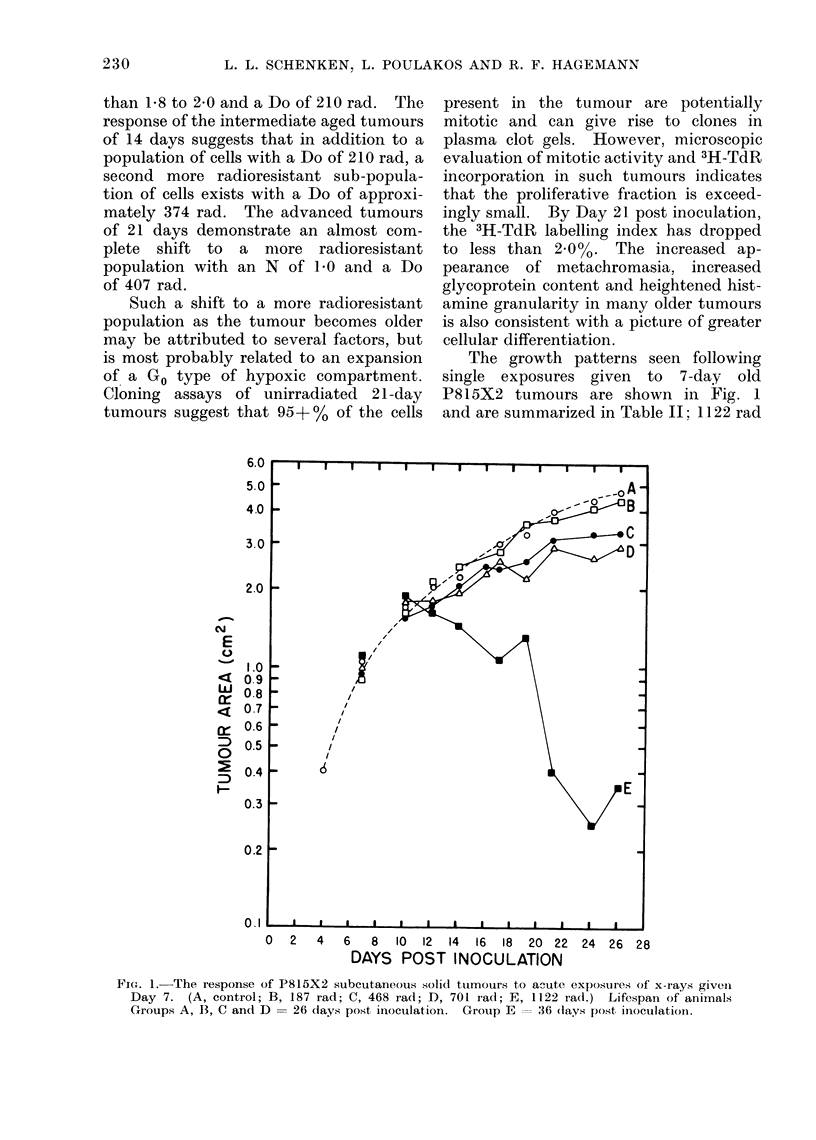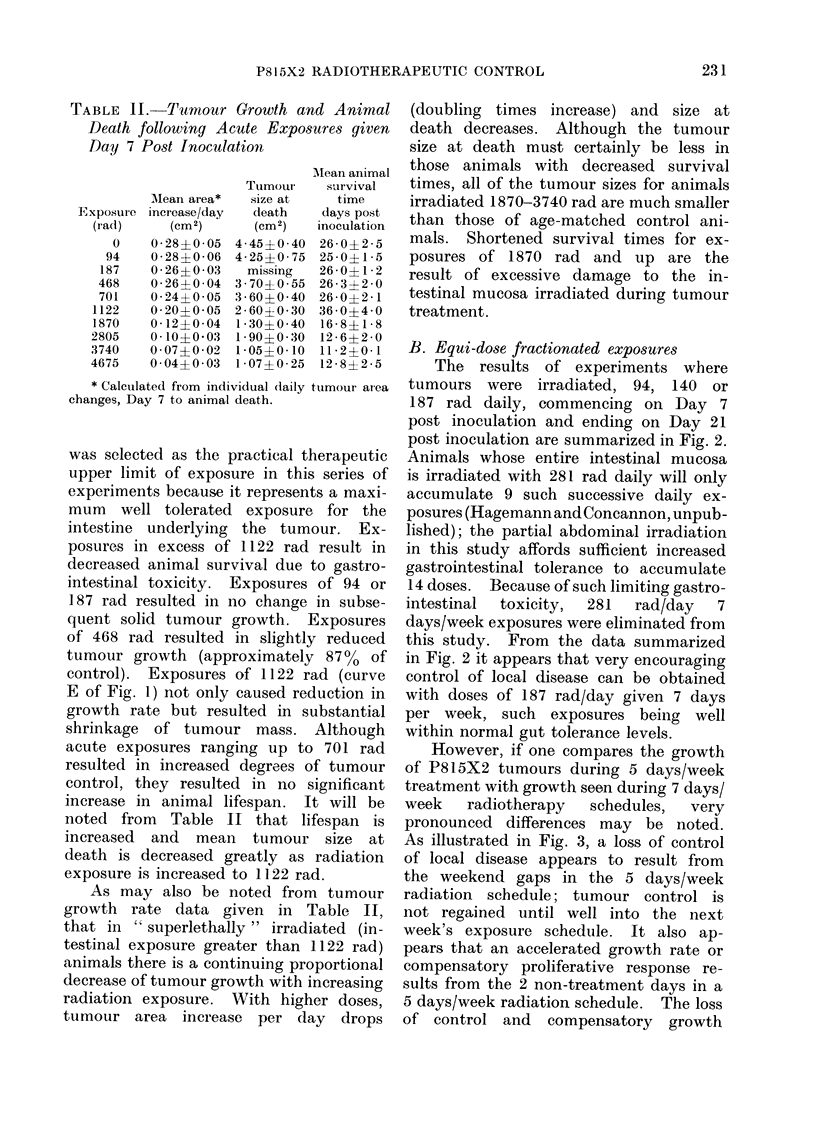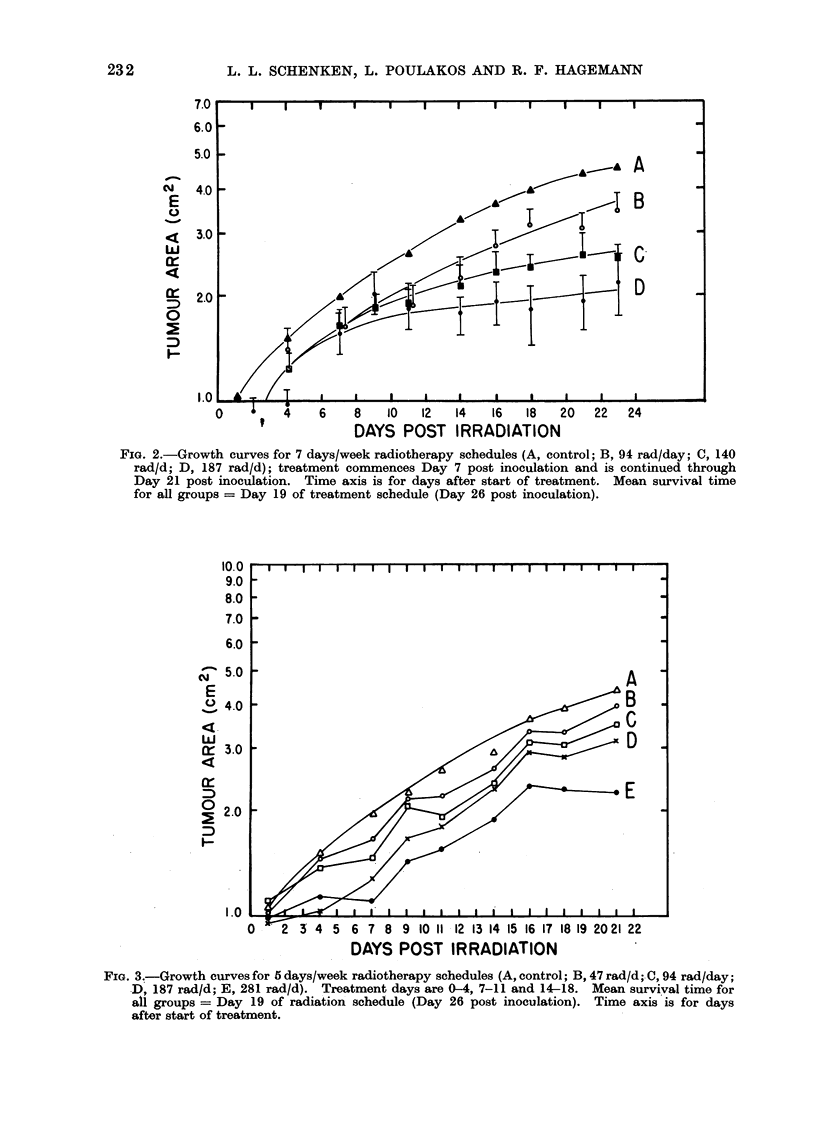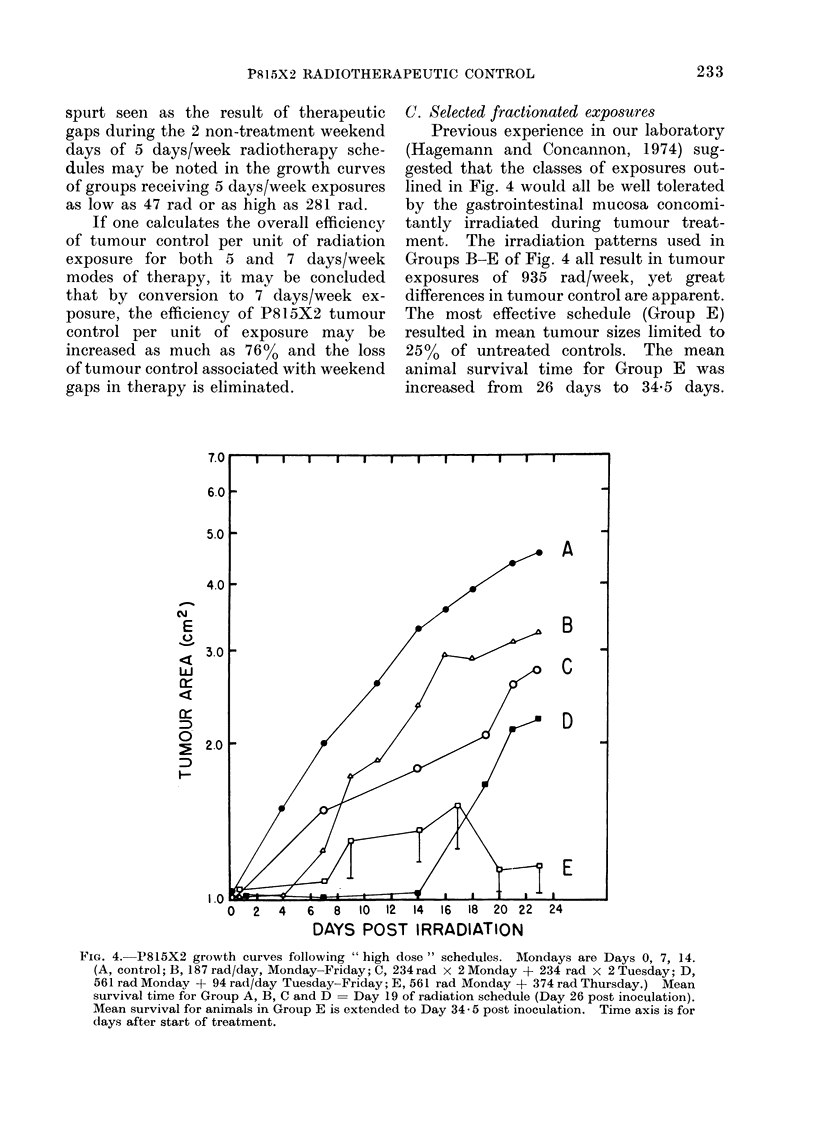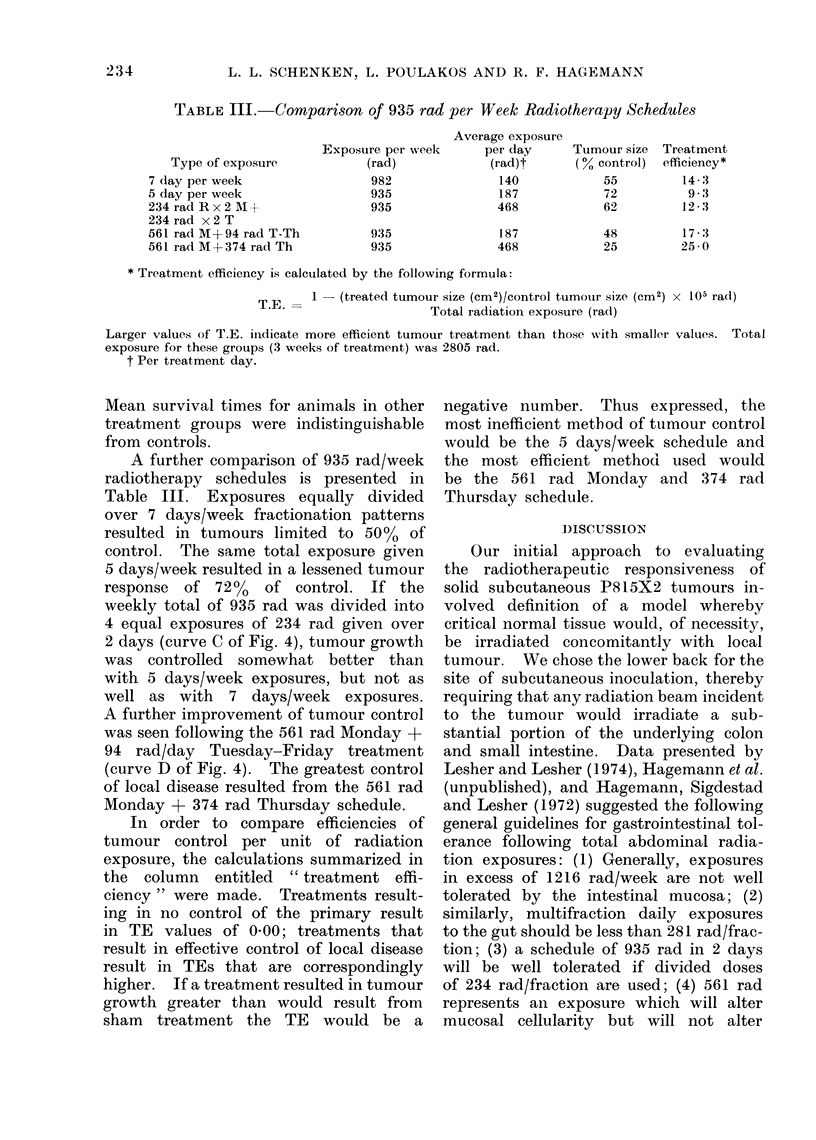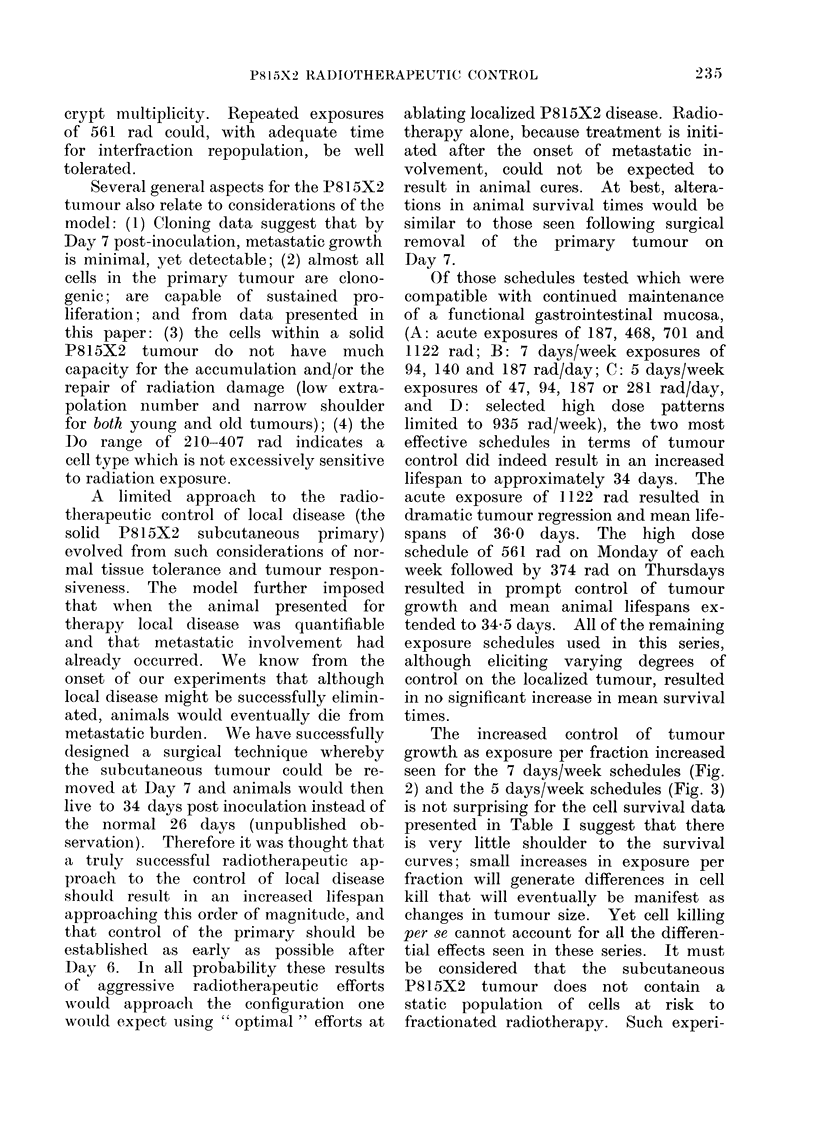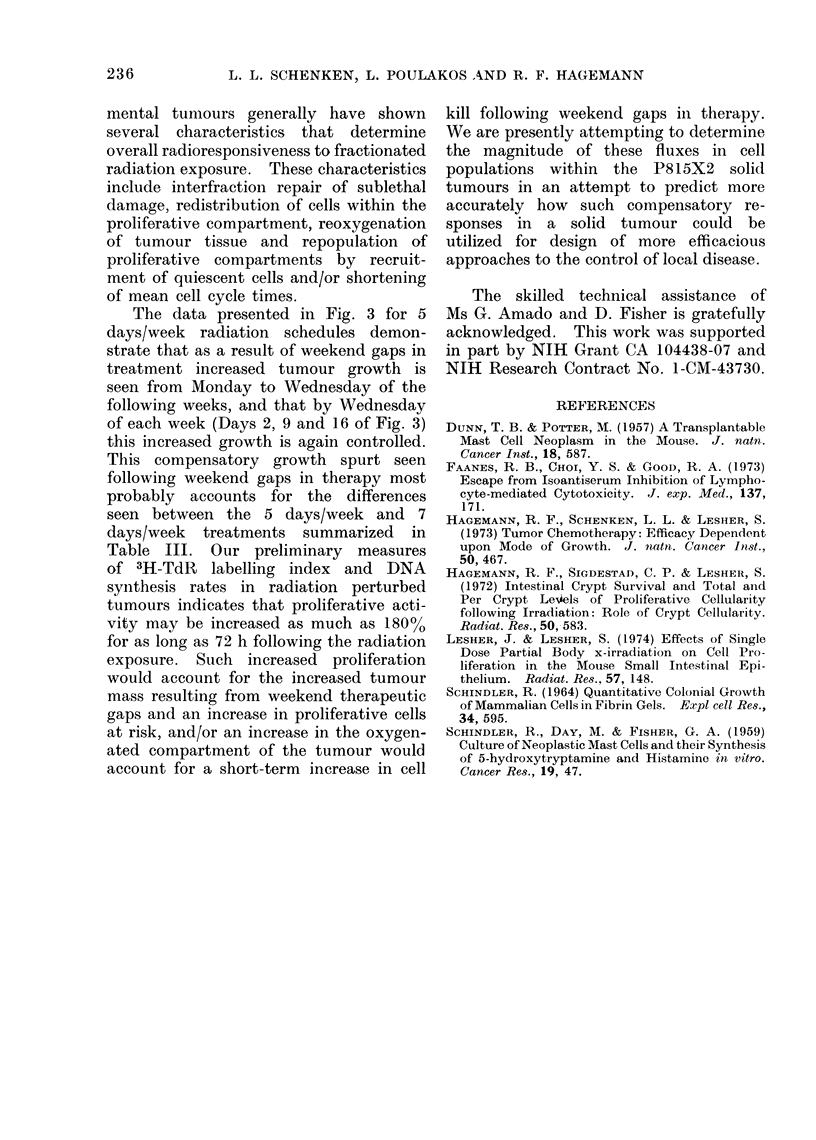# Responses of an experimental solid tumour to irradiation: A comparison of modes of fractionation.

**DOI:** 10.1038/bjc.1975.29

**Published:** 1975-02

**Authors:** L. L. Schenken, L. Poulakos, R. F. Hagemann

## Abstract

Several radiotherapeutic schedules compatible with continued structural-functional integrity of the gastrointestinal (GI) mucosa were compared utilizing the P815X2 murine mastocytoma grown as a solid subcutaneous tumour. Both the tumour and underlying normal tissues were irradiated during the treatments. The tumour exhibited a Do that increased from 210 rad to 397 rad as the tumour aged and in all instances demonstrated minimal shoulders in survival curves. In spite of a relative radioresistance of cells within the solid tumour, quite effective control of localized disease could be accomplished with radiotherapy schemes compatible with GI tolerance limits. Schedules evaluated utilizing this model included acute exposures to 1122 rad, daily exposure to 187 rad, 5 days/week exposures to 281 rad, twice weekly exposures (561 rad on Mondays and 374 rad on Thursdays) and a high dose, two fractions per day, schedule. Tumours were followed for changes in growth patterns during these schedules. Efficacy of tumour control was determined and schedules were compared on this basis. Aggressive radiotherapy approaching the tolerance limits of any of the fractionation schemes proved most effective.


*Br. J. Cancer* (1975) **31**, 228

### RESPONSES OF AN EXPERIMENTAL SOLID TUMOUR TO IRRADIATION: A COMPARISON OF MODES OF FRACTIONATION

L. L. SCHENKEN, L. POULAKOS AND R. F. HAGEMANN

*From the Clinical Radiation Therapy Research Center, Division of Oncology/Radiotherapy, Allegheny General Hospital,* 320 *East North Avenue, Pittsburgh, Pennsylvania* 15212

Received 20 August 1974. Accepted 1 November 1974

**Summary.—Several radiotherapeutic schedules compatible with continued structural–functional integrity of the gastrointestinal (GI) mucosa were compared utilizing the P815X2 murine mastocytoma grown as a solid subcutaneous tumour. Both the tumour and underlying normal tissues were irradiated during the treatments. The tumour exhibited a Do that increased from 210 rad to 397 rad as the tumour aged and in all instances demonstrated minimal shoulders in survival curves. In spite of a relative radioresistance of cells within the solid tumour, quite effective control of localized disease could be accomplished with radiotherapy schemes compatible with GI tolerance limits.**

**Schedules evaluated utilizing this model included acute exposures to 1122 rad, daily exposure to 187 rad, 5 days/week exposures to 281 rad, twice weekly exposures (561 rad on Mondays and 374 rad on Thursdays) and a high dose, two fractions per day, schedule. Tumours were followed for changes in growth patterns during these schedules. Efficacy of tumour control was determined and schedules were compared on this basis. Aggressive radiotherapy approaching the tolerance limits of any of the fractionation schemes proved most effective.**

IN THE present communication we evaluate the efficacy of several radiotherapeutic approaches used for the treatment of localized subcutaneous tumours and report results of fractionated exposure schedules that are limited by normal tissue (underlying gastrointestinal mucosa) tolerance. Seven days per week, 5 days per week, intermittent high dose schedules and acute exposure experiments were selected so that intact, functional gastrointestinal mucosa would be maintained throughout the therapeutic efforts. The P815X2 murine mastocytoma used in these studies demonstrates many characteristics which are advantageous in a model system: The tumour may be grown and evaluated in DBA/2 mice as a solid subcutaneous primary, in metastatic foci or as an ascites tumour. It is responsive to chemotherapeutic (Hagemann, Schenken, and Lesher, 1973; Hagemann, *et al.* unpublished), immunological (Faanes, Choi and Good, 1973) and radiotherapeutic manipulation. The tumour grows in a predictable fashion following subcutaneous implantation, metastasizes to lung, spleen and lymph nodes in an ordered pattern, and demonstrates invasiveness and differentiation patterns similar to many human neoplasms (Schenken, *et al.* unpublished).

### MATERIALS AND METHODS

*Tumour.*—The P815X2 murine mastocytoma used in these studies was originally described by Dunn and Potter (1957). The cells are maintained in our laboratory by serial inoculation of suspension type cultures as described by Schindler, Day and Fisher (1959). Primary tumours were established in DBA/2 mice (Jackson Laboratories, Bar Harbor, Maine) by the subcutaneous (lower back) inoculation of $1 \cdot 0 \times 10^6$ log phase cells from such suspension cultures. By Day 7 post inoculation, tumours have grown to readily measurable size and metastatic involvement is detectable, although not severe.

*Irradiation.*—Partial abdomen irradiation was performed at various times following inoculation of the tumour. A beam incident to the lower back of the animal was used. Although beams were adjusted to include the tumour beds, areas of underlying normal tissue were also purposely irradiated. Fields extended from slightly posterior to the renal beds to a line through the iliofemoral joints. Physical parameters of irradiation were 275 kVcp, 20 mA, 0·5 mm Al, 1·0 mm Cu added filtration, h.v.l. = 1·8 mm Cu, exposure rate 117 rad/min.

*Treatment schedules.*—Animals were inoculated with $1 \cdot 0 \times 10^6$ P815X2 tumour cells on Day 0. On Day 6 post inoculation, groups of 10 animals each were started on the following classes of treatment schedules: A—7 day/week 94, 140 or 187 rad/day; B—5 day/week (Monday–Friday) 47, 94, 187 or 281 rad/day; C—561 rad Monday followed by 94 rad/day Tuesday–Friday; D—561 rad Monday followed by 374 rad Thursday; and E—234 rad, wait 4 h then an additional 234 rad on Monday followed by 234 rad, wait 4 h and a second 234 rad on Tuesday. Several groups of animals were also given varying acute exposures of radiation (up to 1122 rad) on Day 7 post inoculation only. All radiation exposures were given at 9 a.m. on treatment days.

*Primary tumour growth.*—Measurements of tumour length and width (to the nearest mm) were made at daily intervals from Day 7 to Day 21 post inoculation. Tumour area defined as $\pi(\text{length} + \text{width}/4)^2$ was calculated for each animal and mean tumour area $\pm$ s.e. was calculated daily for each experimental group. It has previously been verified by Schenken *et al.* (1974), that tumour area is proportional to tumour volume, mass and cellularity during growth of the solid P815X2 mastocytoma.

In addition to group changes in tumour size, individual animals were followed for daily tumour area changes; daily mean changes of tumour size were computed and overall mean tumour area changes per day were subsequently calculated for each group.

*Cell survival analysis.*—Solid subcutaneous P815X2 tumours 7, 14 and 21 days old were irradiated *in situ* with graded exposures of x-rays to determine the *in vivo* cell survival curves for the tumours of different age. Following radiation exposure, tumours were excised aseptically and single cell suspensions of tumour cells prepared. Cells were assayed for clonogenicity (survival) by using an *in vitro* clot cloning assay. Details of the procedure have been previously described by Schindler (1964).

### RESULTS

### *A. Acute exposure*

Table I summarizes the results of plasma clot cloning assays performed following *in vivo* irradiations of solid tumours 7, 14 and 21 days old. The 7-day tumour demonstrates a response typical of a homogenous population with an extrapolation number, N, no greater

TABLE I.—*P815X2* in vivo *Cell Survival for P815X2 Tumours* 7, 14 *and* 21 *Days Post Inoculation*

| Exposure (rad) | CE*: 7 day tumour (%) | CE: 14 day tumour (%) | CE: 21 day tumour (%) |
|---|---|---|---|
| 0 | 95±10 | 100±5 | 100±6 |
| 94 | 75±15 | 67±4 | 79±4 |
| 187 | 50±7 | 46±9 | 56±3 |
| 468 | 9±2 | 16±5 | 33±4 |
| 748 | 1·5±0·5 | 8±1·5 | 15±3 |
| 935 | 2·0±0·7 | 3·5±1·2 | 5±2 |
| 1496 | 0·25±0·3 | 1·6±0·4 | 2±0·9 |
| | | (Subpopulations) | |
| N | <1·9 | A: 1·0 B: ? | 1·0 |
| Do | 210 rad | A: 220 rad B: 397 rad | 397 rad |

* Cloning efficiency.

than 1·8 to 2·0 and a Do of 210 rad. The response of the intermediate aged tumours of 14 days suggests that in addition to a population of cells with a Do of 210 rad, a second more radioresistant sub-population of cells exists with a Do of approximately 374 rad. The advanced tumours of 21 days demonstrate an almost complete shift to a more radioresistant population with an N of 1·0 and a Do of 407 rad.

Such a shift to a more radioresistant population as the tumour becomes older may be attributed to several factors, but is most probably related to an expansion of a $G_0$ type of hypoxic compartment. Cloning assays of unirradiated 21-day tumours suggest that 95+% of the cells present in the tumour are potentially mitotic and can give rise to clones in plasma clot gels. However, microscopic evaluation of mitotic activity and $^3$H-TdR incorporation in such tumours indicates that the proliferative fraction is exceedingly small. By Day 21 post inoculation, the $^3$H-TdR labelling index has dropped to less than 2·0%. The increased appearance of metachromasia, increased glycoprotein content and heightened histamine granularity in many older tumours is also consistent with a picture of greater cellular differentiation.

The growth patterns seen following single exposures given to 7-day old P815X2 tumours are shown in Fig. 1 and are summarized in Table II; 1122 rad

FIG. 1.—The response of P815X2 subcutaneous solid tumours to acute exposures of x-rays given Day 7. (A, control; B, 187 rad; C, 468 rad; D, 701 rad; E, 1122 rad.) Lifespan of animals Groups A, B, C and D = 26 days post inoculation. Group E = 36 days post inoculation.

TABLE II.—*Tumour Growth and Animal Death following Acute Exposures given Day 7 Post Inoculation*

| Exposure (rad) | Mean area* increase/day ($cm^2$) | Tumour size at death ($cm^2$) | Mean animal survival time days post inoculation |
|---|---|---|---|
| 0 | 0·28±0·05 | 4·45±0·40 | 26·0±2·5 |
| 94 | 0·28±0·06 | 4·25±0·75 | 25·0±1·5 |
| 187 | 0·26±0·03 | missing | 26·0±1·2 |
| 468 | 0·26±0·04 | 3·70±0·55 | 26·3±2·0 |
| 701 | 0·24±0·05 | 3·60±0·40 | 26·0±2·1 |
| 1122 | 0·20±0·05 | 2·60±0·30 | 36·0±4·0 |
| 1870 | 0·12±0·04 | 1·30±0·40 | 16·8±1·8 |
| 2805 | 0·10±0·03 | 1·90±0·30 | 12·6±2·0 |
| 3740 | 0·07±0·02 | 1·05±0·10 | 11·2±0·1 |
| 4675 | 0·04±0·03 | 1·07±0·25 | 12·8±2·5 |

* Calculated from individual daily tumour area changes, Day 7 to animal death.

was selected as the practical therapeutic upper limit of exposure in this series of experiments because it represents a maximum well tolerated exposure for the intestine underlying the tumour. Exposures in excess of 1122 rad result in decreased animal survival due to gastrointestinal toxicity. Exposures of 94 or 187 rad resulted in no change in subsequent solid tumour growth. Exposures of 468 rad resulted in slightly reduced tumour growth (approximately 87% of control). Exposures of 1122 rad (curve E of Fig. 1) not only caused reduction in growth rate but resulted in substantial shrinkage of tumour mass. Although acute exposures ranging up to 701 rad resulted in increased degrees of tumour control, they resulted in no significant increase in animal lifespan. It will be noted from Table II that lifespan is increased and mean tumour size at death is decreased greatly as radiation exposure is increased to 1122 rad.

As may also be noted from tumour growth rate data given in Table II, that in "superlethally" irradiated (intestinal exposure greater than 1122 rad) animals there is a continuing proportional decrease of tumour growth with increasing radiation exposure. With higher doses, tumour area increase per day drops (doubling times increase) and size at death decreases. Although the tumour size at death must certainly be less in those animals with decreased survival times, all of the tumour sizes for animals irradiated 1870–3740 rad are much smaller than those of age-matched control animals. Shortened survival times for exposures of 1870 rad and up are the result of excessive damage to the intestinal mucosa irradiated during tumour treatment.

*B. Equi-dose fractionated exposures*

The results of experiments where tumours were irradiated, 94, 140 or 187 rad daily, commencing on Day 7 post inoculation and ending on Day 21 post inoculation are summarized in Fig. 2. Animals whose entire intestinal mucosa is irradiated with 281 rad daily will only accumulate 9 such successive daily exposures (Hagemann and Concannon, unpublished); the partial abdominal irradiation in this study affords sufficient increased gastrointestinal tolerance to accumulate 14 doses. Because of such limiting gastrointestinal toxicity, 281 rad/day 7 days/week exposures were eliminated from this study. From the data summarized in Fig. 2 it appears that very encouraging control of local disease can be obtained with doses of 187 rad/day given 7 days per week, such exposures being well within normal gut tolerance levels.

However, if one compares the growth of P815X2 tumours during 5 days/week treatment with growth seen during 7 days/week radiotherapy schedules, very pronounced differences may be noted. As illustrated in Fig. 3, a loss of control of local disease appears to result from the weekend gaps in the 5 days/week radiation schedule; tumour control is not regained until well into the next week's exposure schedule. It also appears that an accelerated growth rate or compensatory proliferative response results from the 2 non-treatment days in a 5 days/week radiation schedule. The loss of control and compensatory growth

FIG. 2.—Growth curves for 7 days/week radiotherapy schedules (A, control; B, 94 rad/day; C, 140 rad/d; D, 187 rad/d); treatment commences Day 7 post inoculation and is continued through Day 21 post inoculation. Time axis is for days after start of treatment. Mean survival time for all groups = Day 19 of treatment schedule (Day 26 post inoculation).

FIG. 3.—Growth curves for 5 days/week radiotherapy schedules (A, control; B, 47 rad/d; C, 94 rad/day; D, 187 rad/d; E, 281 rad/d). Treatment days are 0–4, 7–11 and 14–18. Mean survival time for all groups = Day 19 of radiation schedule (Day 26 post inoculation). Time axis is for days after start of treatment.

spurt seen as the result of therapeutic gaps during the 2 non-treatment weekend days of 5 days/week radiotherapy schedules may be noted in the growth curves of groups receiving 5 days/week exposures as low as 47 rad or as high as 281 rad.

If one calculates the overall efficiency of tumour control per unit of radiation exposure for both 5 and 7 days/week modes of therapy, it may be concluded that by conversion to 7 days/week exposure, the efficiency of P815X2 tumour control per unit of exposure may be increased as much as 76% and the loss of tumour control associated with weekend gaps in therapy is eliminated.

*C. Selected fractionated exposures*

Previous experience in our laboratory (Hagemann and Concannon, 1974) suggested that the classes of exposures outlined in Fig. 4 would all be well tolerated by the gastrointestinal mucosa concomitantly irradiated during tumour treatment. The irradiation patterns used in Groups B–E of Fig. 4 all result in tumour exposures of 935 rad/week, yet great differences in tumour control are apparent. The most effective schedule (Group E) resulted in mean tumour sizes limited to 25% of untreated controls. The mean animal survival time for Group E was increased from 26 days to 34·5 days.

FIG. 4.—P815X2 growth curves following " high dose " schedules. Mondays are Days 0, 7, 14. (A, control; B, 187 rad/day, Monday–Friday; C, 234 rad × 2 Monday + 234 rad × 2 Tuesday; D, 561 rad Monday + 94 rad/day Tuesday–Friday; E, 561 rad Monday + 374 rad Thursday.) Mean survival time for Group A, B, C and D = Day 19 of radiation schedule (Day 26 post inoculation). Mean survival for animals in Group E is extended to Day 34·5 post inoculation. Time axis is for days after start of treatment.

TABLE III.—*Comparison of 935 rad per Week Radiotherapy Schedules*

| Type of exposure | Exposure per week (rad) | Average exposure per day (rad)† | Tumour size (% control) | Treatment efficiency* |
|---|---|---|---|---|
| 7 day per week | 982 | 140 | 55 | 14·3 |
| 5 day per week | 935 | 187 | 72 | 9·3 |
| 234 rad R × 2 M + 234 rad × 2 T | 935 | 468 | 62 | 12·3 |
| 561 rad M + 94 rad T-Th | 935 | 187 | 48 | 17·3 |
| 561 rad M + 374 rad Th | 935 | 468 | 25 | 25·0 |

* Treatment efficiency is calculated by the following formula:

$$\text{T.E.} = \frac{1 - (\text{treated tumour size (cm}^2)/\text{control tumour size (cm}^2) \times 10^5 \text{ rad})}{\text{Total radiation exposure (rad)}}$$

Larger values of T.E. indicate more efficient tumour treatment than those with smaller values. Total exposure for these groups (3 weeks of treatment) was 2805 rad.

† Per treatment day.

Mean survival times for animals in other treatment groups were indistinguishable from controls.

A further comparison of 935 rad/week radiotherapy schedules is presented in Table III. Exposures equally divided over 7 days/week fractionation patterns resulted in tumours limited to 50% of control. The same total exposure given 5 days/week resulted in a lessened tumour response of 72% of control. If the weekly total of 935 rad was divided into 4 equal exposures of 234 rad given over 2 days (curve C of Fig. 4), tumour growth was controlled somewhat better than with 5 days/week exposures, but not as well as with 7 days/week exposures. A further improvement of tumour control was seen following the 561 rad Monday + 94 rad/day Tuesday–Friday treatment (curve D of Fig. 4). The greatest control of local disease resulted from the 561 rad Monday + 374 rad Thursday schedule.

In order to compare efficiencies of tumour control per unit of radiation exposure, the calculations summarized in the column entitled "treatment efficiency" were made. Treatments resulting in no control of the primary result in TE values of 0·00; treatments that result in effective control of local disease result in TEs that are correspondingly higher. If a treatment resulted in tumour growth greater than would result from sham treatment the TE would be a negative number. Thus expressed, the most inefficient method of tumour control would be the 5 days/week schedule and the most efficient method used would be the 561 rad Monday and 374 rad Thursday schedule.

### DISCUSSION

Our initial approach to evaluating the radiotherapeutic responsiveness of solid subcutaneous P815X2 tumours involved definition of a model whereby critical normal tissue would, of necessity, be irradiated concomitantly with local tumour. We chose the lower back for the site of subcutaneous inoculation, thereby requiring that any radiation beam incident to the tumour would irradiate a substantial portion of the underlying colon and small intestine. Data presented by Lesher and Lesher (1974), Hagemann *et al.* (unpublished), and Hagemann, Sigdestad and Lesher (1972) suggested the following general guidelines for gastrointestinal tolerance following total abdominal radiation exposures: (1) Generally, exposures in excess of 1216 rad/week are not well tolerated by the intestinal mucosa; (2) similarly, multifraction daily exposures to the gut should be less than 281 rad/fraction; (3) a schedule of 935 rad in 2 days will be well tolerated if divided doses of 234 rad/fraction are used; (4) 561 rad represents an exposure which will alter mucosal cellularity but will not alter crypt multiplicity. Repeated exposures of 561 rad could, with adequate time for interfraction repopulation, be well tolerated.

Several general aspects for the P815X2 tumour also relate to considerations of the model: (1) Cloning data suggest that by Day 7 post-inoculation, metastatic growth is minimal, yet detectable; (2) almost all cells in the primary tumour are clonogenic; are capable of sustained proliferation; and from data presented in this paper: (3) the cells within a solid P815X2 tumour do not have much capacity for the accumulation and/or the repair of radiation damage (low extrapolation number and narrow shoulder for *both* young and old tumours); (4) the Do range of 210–407 rad indicates a cell type which is not excessively sensitive to radiation exposure.

A limited approach to the radiotherapeutic control of local disease (the solid P815X2 subcutaneous primary) evolved from such considerations of normal tissue tolerance and tumour responsiveness. The model further imposed that when the animal presented for therapy local disease was quantifiable and that metastatic involvement had already occurred. We know from the onset of our experiments that although local disease might be successfully eliminated, animals would eventually die from metastatic burden. We have successfully designed a surgical technique whereby the subcutaneous tumour could be removed at Day 7 and animals would then live to 34 days post inoculation instead of the normal 26 days (unpublished observation). Therefore it was thought that a truly successful radiotherapeutic approach to the control of local disease should result in an increased lifespan approaching this order of magnitude, and that control of the primary should be established as early as possible after Day 6. In all probability these results of aggressive radiotherapeutic efforts would approach the configuration one would expect using " optimal " efforts at ablating localized P815X2 disease. Radiotherapy alone, because treatment is initiated after the onset of metastatic involvement, could not be expected to result in animal cures. At best, alterations in animal survival times would be similar to those seen following surgical removal of the primary tumour on Day 7.

Of those schedules tested which were compatible with continued maintenance of a functional gastrointestinal mucosa, (A: acute exposures of 187, 468, 701 and 1122 rad; B: 7 days/week exposures of 94, 140 and 187 rad/day; C: 5 days/week exposures of 47, 94, 187 or 281 rad/day, and D: selected high dose patterns limited to 935 rad/week), the two most effective schedules in terms of tumour control did indeed result in an increased lifespan to approximately 34 days. The acute exposure of 1122 rad resulted in dramatic tumour regression and mean lifespans of 36·0 days. The high dose schedule of 561 rad on Monday of each week followed by 374 rad on Thursdays resulted in prompt control of tumour growth and mean animal lifespans extended to 34·5 days. All of the remaining exposure schedules used in this series, although eliciting varying degrees of control on the localized tumour, resulted in no significant increase in mean survival times.

The increased control of tumour growth as exposure per fraction increased seen for the 7 days/week schedules (Fig. 2) and the 5 days/week schedules (Fig. 3) is not surprising for the cell survival data presented in Table I suggest that there is very little shoulder to the survival curves; small increases in exposure per fraction will generate differences in cell kill that will eventually be manifest as changes in tumour size. Yet cell killing *per se* cannot account for all the differential effects seen in these series. It must be considered that the subcutaneous P815X2 tumour does not contain a static population of cells at risk to fractionated radiotherapy. Such experimental tumours generally have shown several characteristics that determine overall radioresponsiveness to fractionated radiation exposure. These characteristics include interfraction repair of sublethal damage, redistribution of cells within the proliferative compartment, reoxygenation of tumour tissue and repopulation of proliferative compartments by recruitment of quiescent cells and/or shortening of mean cell cycle times.

The data presented in Fig. 3 for 5 days/week radiation schedules demonstrate that as a result of weekend gaps in treatment increased tumour growth is seen from Monday to Wednesday of the following weeks, and that by Wednesday of each week (Days 2, 9 and 16 of Fig. 3) this increased growth is again controlled. This compensatory growth spurt seen following weekend gaps in therapy most probably accounts for the differences seen between the 5 days/week and 7 days/week treatments summarized in Table III. Our preliminary measures of $^{3}$H-TdR labelling index and DNA synthesis rates in radiation perturbed tumours indicates that proliferative activity may be increased as much as 180% for as long as 72 h following the radiation exposure. Such increased proliferation would account for the increased tumour mass resulting from weekend therapeutic gaps and an increase in proliferative cells at risk, and/or an increase in the oxygenated compartment of the tumour would account for a short-term increase in cell kill following weekend gaps in therapy. We are presently attempting to determine the magnitude of these fluxes in cell populations within the P815X2 solid tumours in an attempt to predict more accurately how such compensatory responses in a solid tumour could be utilized for design of more efficacious approaches to the control of local disease.

The skilled technical assistance of Ms G. Amado and D. Fisher is gratefully acknowledged. This work was supported in part by NIH Grant CA 104438-07 and NIH Research Contract No. 1-CM-43730.

### REFERENCES

DUNN, T. B. & POTTER, M. (1957) A Transplantable Mast Cell Neoplasm in the Mouse. *J. natn. Cancer Inst.*, **18**, 587.

FAANES, R. B., CHOI, Y. S. & GOOD, R. A. (1973) Escape from Isoantiserum Inhibition of Lymphocyte-mediated Cytotoxicity. *J. exp. Med.*, **137**, 171.

HAGEMANN, R. F., SCHENKEN, L. L. & LESHER, S. (1973) Tumor Chemotherapy: Efficacy Dependent upon Mode of Growth. *J. natn. Cancer Inst.*, **50**, 467.

HAGEMANN, R. F., SIGDESTAD, C. P. & LESHER, S. (1972) Intestinal Crypt Survival and Total and Per Crypt Levels of Proliferative Cellularity following Irradiation: Role of Crypt Cellularity. *Radiat. Res.*, **50**, 583.

LESHER, J. & LESHER, S. (1974) Effects of Single Dose Partial Body x-irradiation on Cell Proliferation in the Mouse Small Intestinal Epithelium. *Radiat. Res.*, **57**, 148.

SCHINDLER, R. (1964) Quantitative Colonial Growth of Mammalian Cells in Fibrin Gels. *Expl cell Res.*, **34**, 595.

SCHINDLER, R., DAY, M. & FISHER, G. A. (1959) Culture of Neoplastic Mast Cells and their Synthesis of 5-hydroxytryptamine and Histamine *in vitro*. *Cancer Res.*, **19**, 47.